# Discovery and Characterisation of Dual Inhibitors of Tryptophan 2,3-Dioxygenase (TDO2) and Indoleamine 2,3-Dioxygenase 1 (IDO1) Using Virtual Screening

**DOI:** 10.3390/molecules24234346

**Published:** 2019-11-28

**Authors:** Suat Sari, Petr Tomek, Euphemia Leung, Jóhannes Reynisson

**Affiliations:** 1 Hacettepe University, Faculty of Pharmacy, Department of Pharmaceutical Chemistry, Ankara 06100, Turkey; suat1039@gmail.com; 2 Auckland Cancer Society Research Centre, University of Auckland, Grafton, 1023 Auckland, New Zealand; p.tomek@auckland.ac.nz; 3 School of Pharmacy and Bioengineering, Keele University, Hornbeam building, Staffordshire ST5 5BG, UK

**Keywords:** kynurenine, haem-containing dioxygenases, cancer immunotherapy, ligand and pharmacophore-based screening, molecular modelling, chemical space

## Abstract

Cancers express tryptophan catabolising enzymes indoleamine 2,3-dioxygenase 1 (IDO1) and tryptophan 2,3-dioxygenase (TDO2) to produce immunosuppressive tryptophan metabolites that undermine patients’ immune systems, leading to poor disease outcomes. Both enzymes are validated targets for cancer immunotherapy but there is a paucity of potent TDO2 and dual IDO1/TDO2 inhibitors. To identify novel dual IDO1/TDO2 scaffolds, 3D shape similarity and pharmacophore in silico screening was conducted using TDO2 as a model for both systems. The obtained hits were tested in cancer cell lines expressing mainly IDO1 (SKOV3—ovarian), predominantly TDO2 (A172—brain), and both IDO1 and TDO2 (BT549—breast). Three virtual screening hits were confirmed as inhibitors (**TD12**, **TD18** and **TD34)**. Dose response experiments showed that **TD34** is the most potent inhibitor capable of blocking both IDO1 and TDO2 activity, with the IC_50_ value for BT549 at 3.42 µM. This work identified new scaffolds able to inhibit both IDO1 and TDO2, thus enriching the collection of dual IDO1/TDO2 inhibitors and providing chemical matter for potential development into future anticancer drugs.

## 1. Introduction

The kynurenine pathway transforms ~95% of the essential amino acid tryptophan into many bioactive metabolites including nicotinamide adenine dinucleotide (NAD^+^), crucial for cellular redox metabolism [1]. Haem-containing enzymes indoleamine 2,3-dioxygenase (IDO1) and tryptophan 2,3-dioxygenase (TDO2) catalyse oxidation of tryptophan into *N*-formyl-kynurenine, the first and rate-limiting step in the kynurenine pathway [2]. *N*-formyl-kynurenine is readily converted into kynurenine inside mammalian cells by kynurenine formamidase [3]. Whilst both IDO1 and TDO2 catalyse the identical biochemical reaction, their physiological roles and protein structures differ substantially. TDO2 is a tetramer (167 kDa) expressed predominantly in the liver to maintain tryptophan homeostasis. In contrast, IDO1 is a monomeric enzyme (37 kDa) essentially absent in most normal tissues but induced during inflammation and mammalian gestation to suppress the immune system [1].

Tryptophan deprivation and the accumulation of tryptophan metabolites such as kynurenine paralyse cancer-killing immune cells and expand the populations of suppressive immune cells in tumour microenvironments, contributing to immune escape and malignancy [4,5]. Many cancers express high levels of TDO2 and IDO1, and high tumoural IDO1 expression is associated with poor patient outcomes [4,6]. The important role of accelerated tryptophan catabolism in escaping immune surveillance makes IDO1 and TDO2 prime targets for development of small molecule drugs to augment cancer immunotherapy. Advanced biochemical assay methodologies [7,8,9] and virtual screening approaches [10,11] have played a key part in discovery of a diverse range of IDO1-specific inhibitors [1,12,13]. Two IDO1 inhibitors, Epacadostat (Incyte Corporation) and Linrodostat (Bristol-Myers-Squibb), have reached Phase 3 clinical trials [14,15,16].

Despite promising results in early clinical trials [17], in phase 3 trial ECHO-301, the combination of Epacadostat and PD-1 inhibitor Pembrolizumab did not provide a large cohort of advanced melanoma patients with any additional survival benefit compared to Pembrolizumab alone [18]. A potential explanation for this unfavourable outcome is the expression of both IDO1 and TDO2 in the patients’ tumours [19]. In this situation, an IDO1-specific inhibitor such as Epacadostat cannot completely block the production of the immunosuppressive tryptophan catabolites because it inhibits TDO2 weakly. Hence, the combination of IDO1- and TDO2-specific inhibitors or administration of dual IDO1/TDO2 inhibitors are promising strategies being explored to more completely silence the immunosuppressive kynurenine pathway. However, a relative dearth of potent TDO2-specific [1,15,20] and dual IDO1/TDO2 inhibitors limits advances in this area [21,22,23].

Structure-based virtual screening is a well-established method to identify hit matter for development of lead- and drug-candidates when a protein structure is available [24,25,26]. Virtual hits need to be tested in a biological assay verifying their affinity for the target of interest. Kynurenine production can be conveniently monitored in a cell-based assay [27,28] and the crystal structures of TDO2 and IDO1 are available [29,30,31]. Herein, we applied the virtual screening technique in conjunction with the cell based kynurenine assay to identify TDO2-specific and dual IDO1/TDO2 inhibitors with potential for further development into drug candidates for cancer immunotherapy.

## 2. Results and Discussion

### 2.1. Validation of Virtual Screening Methods

In order to gauge the prediction power of the virtual screening methodologies available a test set of active and inactive compounds was compiled. Eighty (80) known TDO2 inhibitors were obtained from ChEMBL collection [32], and from the literature [33], thirty-four (34) were defined as active (IC_50_ ≤ 10 µM) and 46 as inactive ligands (IC_50_ > 100 µM). The ligands active in the region between these classifications (10–100 µM) were unfortunately not available in the literature. TDO2 was used as a model for both systems, the rational being that the same substrates are metabolised into the same products by both enzymes. An accumulation curve, which shows the number of screened compounds over the number of actives found in the collection, was plotted for each virtual screening method and compared to an ideal curve. An R_AUC_ value $\left(\frac{AUC_{experimental}}{AUC_{ideal}}\right)$ was computed to illustrate the comparison numerically. An R_AUC_ value of 1.0 reflects ideal performance but a value of 0.5 infers no enrichment. Thus, the accumulation curve of a good-performing method looks similar to the ideal curve whilst a diagonal line is expected for a method with no prediction power.

### 2.2. Molecular Docking

**Figure 1:**
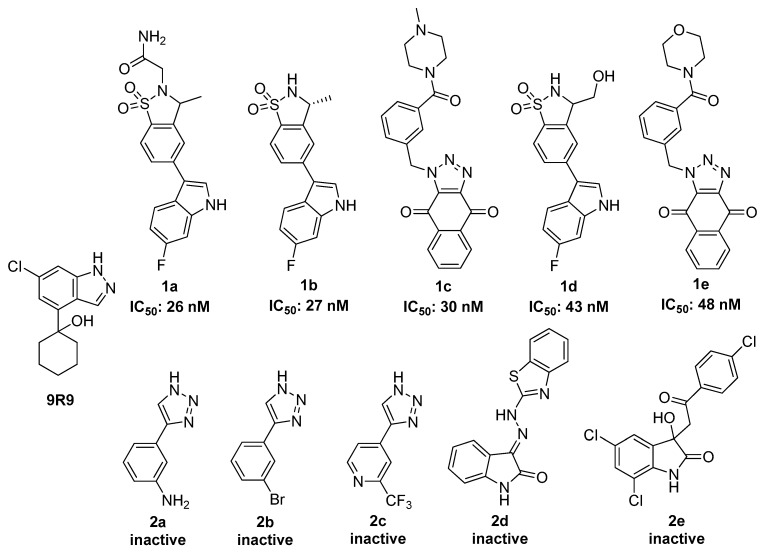
The molecular structures of **9R9** and some of the tryptophan 2,3-dioxygenase (TDO2) active (**1a**–**1e**) and inactive (**2a**–**2e**) compounds in the test library. The complete library is given in the Supplementary Material, Figure S1.

**Figure 2:**
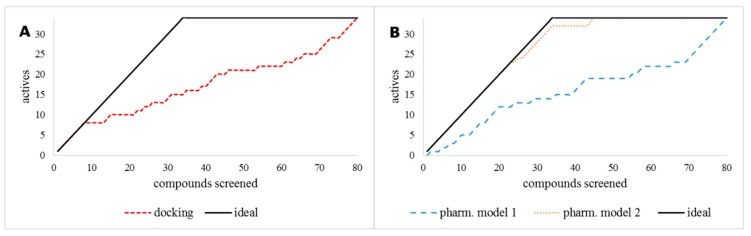
Accumulation curves for (**A**) the molecular docking method and (**B**) pharmacophore models 1 and 2, compared to the ideal curve (black solid line). Whilst (**A**) indicates poor enrichment, pharmacophore model 2 (orange dots) in (**B**) affords excellent enrichment with R_AUC_ value of 0.98.

Inhibitor 1-(6-chloro-1*H*-indazol-4-yl)cyclohexan-1-ol (**9R9**, Figure 1) was re-docked to the TDO2 crystal structure; the predicted pose obtained was very similar to the co-crystallized conformation (root-mean-square deviation (RMSD) 0.59 Å) demonstrating the prediction power of the method. However, when docking scores of the test library were plotted against their experimental IC_50_ (concentration leading to 50% inhibition of activity), the result showed a poor accumulation curve approximating a diagonal line with an R_AUC_ value of only 0.64 (Figure 2).

### 2.3. Pharmacophore Modelling

Two pharmacophore based methods were tested, an e-pharmacophore hypothesis derived from the **9R9**-TDO2 complex (pharmacophore model 1) and a manual pharmacophore hypothesis (pharmacophore model 2) derived from ten active compounds [33,34,35,36] of which five effectively inhibit TDO2 (**1a**–**1e**) and five are inactive (**2a**–**2e**) (Figure 2). The virtual library was screened against these two models. The e-pharmacophore hypothesis yielded poor enrichment (R_AUC_ = 0.59), however the manual model gave nearly ideal enrichment with R_AUC_ = 0.98 as shown in Figure 2B.

### 2.4. 3D Shape Similarity

**Figure 3:**
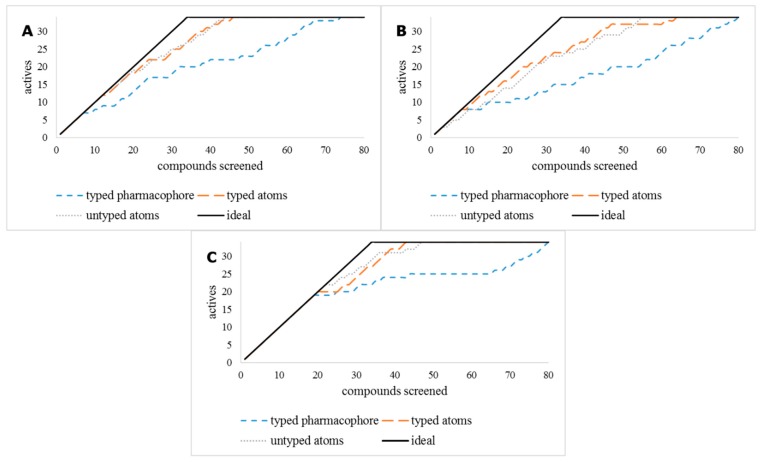
Accumulation curves for the three ligand-based shape screening methods of the query compounds **1a** (**A**), **1b** (**B**) and **1c** (**C**).

Another ligand-based virtual screening method, 3D shape similarity approach, was tested for its ability to predict TDO2 inhibitors. The three most active ligands **1a**, **1b** and **1c** (Figure 1) were selected as query compounds. The virtual library was screened against each using the three methods embedded in Maestro’s Shape Screening panel. Each structure was treated as (a) a collection of pharmacophore sites (typed pharmacophore), (b) van der Waals (vdW) volume rewarding overlaps of the same atom types (typed atoms), and (c) pure vdW volume to treat all atoms the same for defining overlaps (untyped atoms). The accumulation curves were close to the ideal for the three compounds for the typed and un-typed atoms mode (Figure 3) but the R_AUC_ values for the typed pharmacophore mode were considerably lower as shown in Table 1.

**Figure 4:**
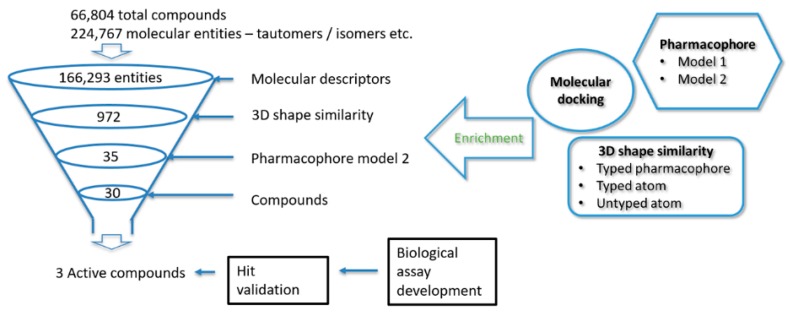
Outline of the ligand- and pharmacophore-based in silico screening cascade and experimental evaluation.

The test library results indicate that the 3D shape screening methods, with typed and untyped atoms using inhibitors **1a** and **1c** as query compounds gave excellent results. It was therefore decided to use these methods to screen the InterBioScreen (IBS) natural product library [37] followed by the pharmacophore model 2. First, the fast 3D shape screening methods were used to quickly reduce the size of the IBS library followed by the more CPU-intensive pharmacophore model 2, as shown in Figure 4.

### 2.5. Virtual Screening of the IBS Library

The IBS natural compounds library (66,804 molecules) was downloaded, optimised and prepared for screening resulting in 224,767 molecular entities including different enantiomers, tautomers and ionization states. The molecular descriptors were generated, the molecular entities < 100 Da and > 700 Da, Log P > 5 and Log S < −6 were removed. Furthermore, molecules with more than two Lipinski’s Rule of Five violations were eliminated [38].

The remaining 166,293 entities were screened for their 3D shape similarity to **1a** and **1c** using the untyped atoms mode. Entities with shape similarity score ≥ 0.85 to **1a** (783 entities/583 compounds) and those with shape similarity score ≥ 0.80 to **1c** (189 entities/164 compounds) were selected for further screening. The resulting compounds were merged and screened against pharmacophore model 2. Entries with Phase screen score ≥ 1.0 (35 entries/30 compounds) were selected for biological evaluation. All the structures are given in Figure S2 in the Supplementary Material section.

### 2.6. Cell-Based Assays for Testing the Inhibitory Activity of Test Compounds

**Figure 5:**
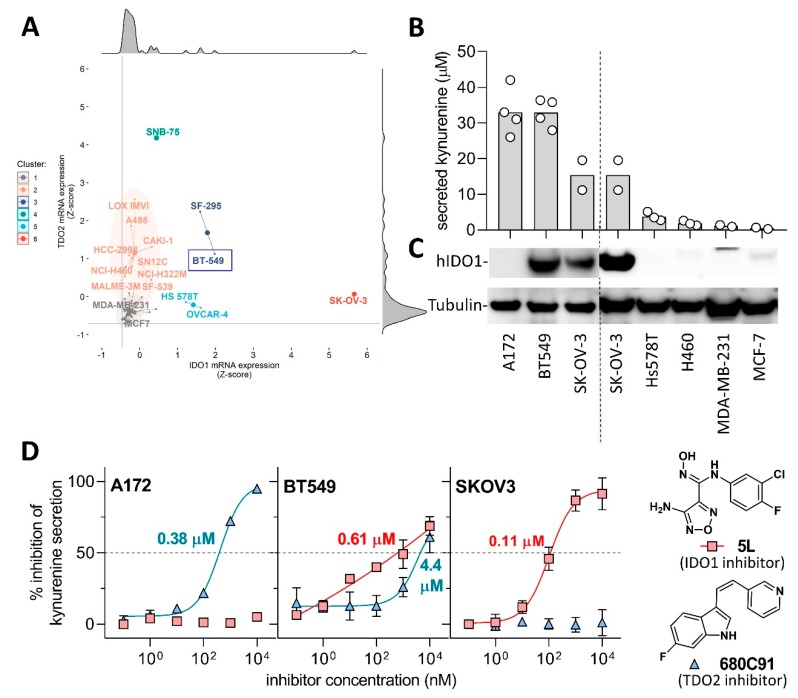
Identification and characterisation of indoleamine 2,3-dioxygenase 1 (IDO1) and tryptophan 2,3- dioxygenase (TDO2) expressing cell lines (**A**) Scatter star plot of normalised TDO2 (y-axis) and IDO1 (x-axis) mRNA expression levels (Z-score) of 60 cancer cell lines from the NCI CellMiner CDB database [41]. Grey horizontal and vertical line demarcates the lowest Z-score values in the dataset. Cell lines are grouped into six clusters based on the unsupervised hierarchical clustering analysis, using Ward.D2 clustering of Manhattan distances. Enlarged points indicate centres of each cluster. Marginal plots represent histograms. (**B**) Secreted kynurenine levels and (**C**) abundance of human IDO1 and α-tubulin (loading control) in cancer cell lines studied. Bar height denotes arithmetic mean of independent experimental measurements represented as white circles. (**D**) Inhibitory activity of reference IDO1 inhibitor **5L** and TDO2 inhibitor **680C91** in A172 glioblastoma, SKOV3 ovarian and BT549 breast cancer cell lines. The values inside the plots indicate IC_50_.

In order to determine the ability of compounds to inhibit kynurenine production in cancer cells and their selectivity for IDO1 and TDO2, we first set out to identify cell lines endogenously expressing only IDO1, only TDO2, and both enzymes simultaneously. It has been reported that the ovarian cancer line SKOV3 expresses exclusively IDO1 [39] and the glioma line A172 expresses predominantly TDO2 [40], but a cell line endogenously expressing both enzymes in the absence of interferon stimulation is yet to be described. To this end, we carried out a meta-analysis of IDO1 and TDO2 transcript abundance available in 60 common cancer lines in the National Cancer Institute database (Figure 5A). Forty-five lines (75%) were essentially devoid of dioxygenase transcripts (Figure 5A, grey cluster 1) consistent with inducible IDO1 expression and the absence of TDO2 in the majority of non-hepatic tissues. The remaining 15 lines (25%) clustered into ten predominantly *TDO2*-expressors (clusters 2 and 4)*,* three IDO1 expressors (clusters 5 and 6) and two dual IDO1/TDO2 expressors (cluster 3).

Consistent with a previous report, SKOV3 expressed the highest levels of IDO1 transcript in this meta-analysis [39]. One of the putative dual expressors (cluster 3), breast cancer line BT549, was available in our laboratory, hence we proceeded to validate its IDO1 abundance and kynurenine production as well as for the positive controls A172 and SKOV3. Furthermore, several negative control cell lines predicted to have minimal or no kynurenine expression in Figure 5A were tested (Figure 5B,C). Due to the paucity of specific anti-TDO2 commercial antibodies, we decided to assess the presence of TDO2 in the cells using a combination of published IDO1-specific and TDO2-specific inhibitors Incyte 5L [42] and 680C91 [43], respectively (see Figure 5D for their chemical structures).

The levels of kynurenine and IDO1 protein produced by the seven lines tested (Figure 5B,C) are markedly consistent with the transcript abundance (Figure 5A). The high TDO2 expressor A172 and the dual IDO1/TDO2 expressor BT549 produced the highest levels of both IDO1 protein and kynurenine whereas IDO1-expressor SKOV3 produced a lower but still substantial amount of kynurenine. The other four lines derived from breast and lung cancers secreted less than 4 µM kynurenine and produced barely detectable amount of IDO1 (Figure 5B,C). Subsequently, the presence of IDO1 and TDO2 in A172, BT549 and SKOV3 was assessed using small-molecule inhibitors (Figure 5D).

The IDO1-specific inhibitor 5L completely inhibited kynurenine production in SKOV3 at 1 µM and provided an IC_50_ value of 10 nM consistent with published results [28,42]. The TDO2-specific inhibitor 680C91 barely affected SKOV3’s kynurenine production, strongly suggesting TDO2 deficiency in the SKOV3 line concordant with the meta-analysis in Figure 5A. A172 showed inversed sensitivity to the two inhibitors tested suggesting IDO1 deficiency. On the other hand, 5L and 680C91 both inhibited kynurenine production in BT549 albeit between 5- and 10-fold less potently indicative of dual expression of IDO1 and TDO2.

In conclusion, these experiments support the presence of only IDO1 in SKOV3 and that of TDO2 in A172, and indicate dual expression of IDO1 and TDO2 in BT549. The A172 and SKOV3 lines served to identify specificity of the inhibitors to IDO1 or TDO2, respectively, and the BT549 line challenged the ability of the compounds to inhibit both dioxygenases simultaneously.

### 2.7. Testing the in Silico Hits for Inhibition of IDO1 and TDO2

**Figure 6:**
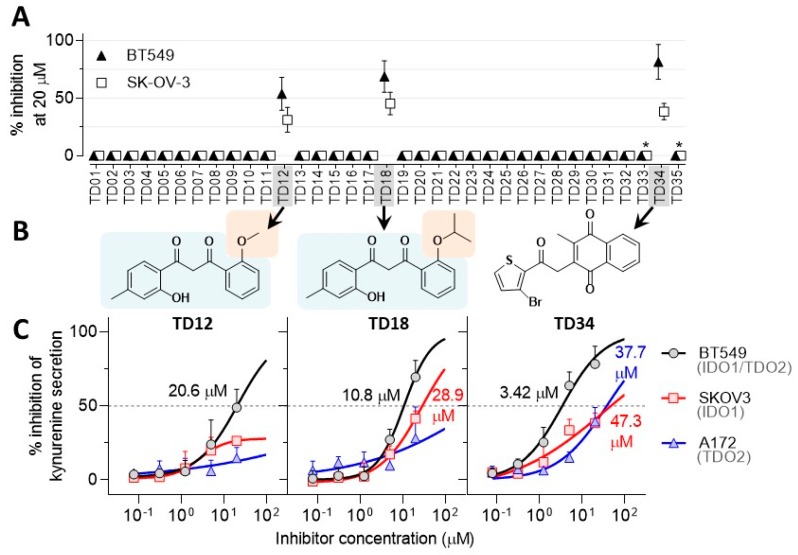
Assessing the inhibitory activity of the virtual hits in cell-based assays. (**A**) Inhibition of kynurenine secretion in SKOV3 and BT549 cell lines by the virtual hits **TD1** to **TD30** and the structural derivative of **TD05**, **TD31** to **TD36** at 20 µM. Asterisks mark compounds toxic to the BT549 and SKOV3 cell lines. (**B**) Chemical structures of the confirmed dioxygenase inhibitors **TD12**, **TD18** and **TD34**. (**C**) Potency of the three confirmed inhibitors in all three cell line models. The values inside the plots are IC_50_ values. Data points are arithmetic means ± standard deviations.

The thirty virtual hits (designated as **TD01** to **TD30**) identified were subsequently tested for their ability to inhibit kynurenine production in SKOV3 and BT549 lines. Two hits (**TD12** and **TD18)** were initially confirmed as kynurenine production inhibitors, indicating a 6% hit rate of the virtual screening. Both compounds inhibited kynurenine secretion to a similar extent (50% ± 20%) (Figure 6A). A higher hit ratio would be expected if a recombinant enzyme assay was used because virtual screening does not take into account cell penetration. In addition, preliminary results indicated that hit **TD05** was active but further investigation did not confirm this finding.

The chemical space surrounding **TD05** and the confirmed inhibitors **TD12** and **TD18** was investigated by conducting similarity and substructure search of commercially available compounds in the eMolecules portal [44]. Derivatives of **TD12** and **TD18** were not commercially available but five analogues of **TD05** were purchased and tested (**TD31**–**35**). Their molecular structures are shown in Figure S3 of the Supplementary Material. Only **TD34** inhibited kynurenine production at 20 µM (Figure 6A) in the absence of cell toxicity. Interestingly, **TD34** shares similarity with previously reported ethylnaphthoquinone derivatives, inhibitors of IDO1 [45]. The molecular structures of the hit compounds **TD12**, **TD18** and **TD34** are shown in Figure 6B. Whilst the inhibitor **TD34** is based on a naphthoquinone core, a class of known IDO1 inhibitors, aromatic diketones **TD12** and **TD18** are novel. **TD18** is a derivative of **TD12** differing only by a larger isopropyl substituent in place of a methyl group. Similar inhibitory activity of both **TD12** and **TD18** suggests that the anisole ring of **TD12** can tolerate larger substituents without significant impact on inhibitory activity. In contrast, substitution of a *meta*-cresol ring of **TD18** by a *para*-cresol ring (**TD28**) results in a complete loss of inhibitory activity (see Figure S2 in the Supplementary Material). This means that the substitution pattern on the phenyl rings is important for the activity of this series and can be further chemically modified to explore the structure activity relationship.

The three confirmed IDO1/TDO2 inhibitors (Figure 6B) were titrated for inhibition of kynurenine secretion in order to determine their potency and selectivity against IDO1 and TDO2 (Figure 6C). **TD34** emerged as the most potent of the three inhibitors. Whilst **TD34** inhibits TDO2 in A172 and IDO1 in SKOV3 relatively weakly (IC_50_ ~ 40 µM), it shows more than 10-fold higher potency in the dual IDO1/TDO2 expressing line BT549 (IC_50_ = 3.45 µM). This appears to be a general trend amongst the three inhibitors; their potency markedly improves in the BT549 line compared to either the A172 or SKOV3 lines. This result indicates that all three inhibitors can bind both IDO1 and TDO2 simultaneously. Even though the two enzymes are structurally different, they metabolise the identical substrate tryptophan, hence the identification of dual IDO1/TDO2 inhibitors is not surprising.

**Figure 7:**
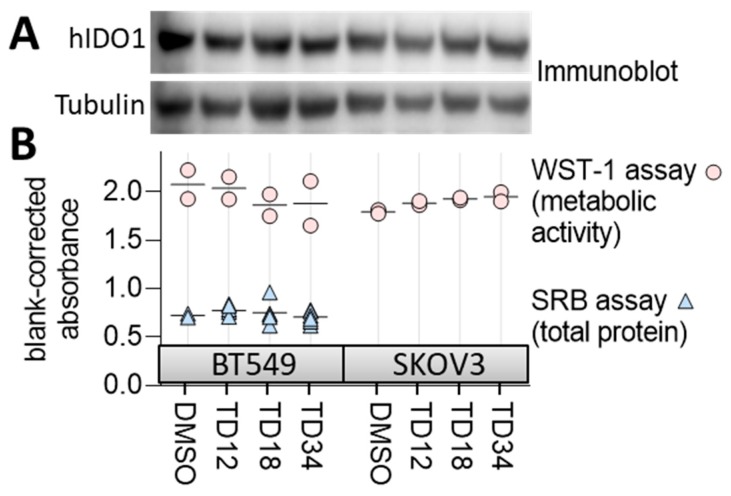
Evaluating specificity and toxicity of the confirmed inhibitors. (**A**) Immunoblot for IDO1 expression in BT549 and SKOV3 lines treated with the inhibitors at 20 µM for 24 h. Tubulin represents a loading control. (**B**) The effect of 20 µM concentration of the inhibitors on cell viability measured by WST-1 assay and cell growth measured by SRB assay.

To confirm that the compounds are nontoxic, we measured changes in metabolic activity and growth of the cells treated with a 20 µM inhibitor dose using WST-1 and SRB assays, respectively (Figure 7). WST-1 tests the bio-reductive potential of the cells as a surrogate of cell fitness whereas SRB stains total protein content of the cells indicative of cell number. None of the three compounds affected total protein content or metabolic activity of the cells when compared to a vehicle treated cell population (Figure 7B) suggesting that the compounds are nontoxic at concentrations inhibitory to at least the BT549 line. None of the three inhibitors interfered with IDO1 protein expression as determined by immunoblotting (Figure 7A). Overall, these observations suggest that the three inhibitors reduce cellular kynurenine secretion by binding to IDO1/TDO2 rather than interfering with the enzyme expression or decreasing cellular fitness. However, due to potential redox activity of the inhibitors, especially **TD34**, the interference with the reductive activation of IDO1/TDO2 cannot be excluded. In order to check the redox stability of the ligands, their ionisation potentials (IP) and electron affinities (EA) were derived using the Density Functional Theory (DFT) method. The IP reflects one-electron oxidation whereas EA one-electron reduction. The established parameters for known drugs is 6.0 to 9.0 eV for IP and −1.5 to 2.0 eV for EA [46]. **TD12** and **TD18** have the same IP value 7.5 eV and again the same EA values of −0.6 eV, comfortably within the 95% confidence limits of known drugs. It can be stated that these two ligands are redox stable. However, **TD34** has an EA value of −2.0 eV, i.e., half an eV lower than the confidence limits for known drugs and is therefore susceptible for reduction. Furthermore, **TD34** does contain a quinone moiety, which is recognised as one of the Pan-assay Interference Compounds (PAINS) [47]. Interestingly, **TD05**, **TD31**, **TD32**, **TD33** and **TD35** also contain quinone but show no or marginal activity. TD35’s IP value is 8.4 eV and within the specified range.

Ideally, the hit compounds should be tested in biochemical assays based on the purified enzymes, verifying their potential and specificity. Nevertheless, the nontoxic nature of the hits indicates that no vital cellular pathways are perturbed but off-target effects cannot be excluded.

### 2.8. Suitability of the Identified Hits for Drug Development—Chemical Space

The mainstream molecular descriptors of molecular weight (MW), number of hydrogen bond donors (HB donor), number of hydrogen bond acceptors (HA acceptor), lipophilicity (Log P), polar surface area (PSA) and number of rotatable bonds (RB) were derived for the hit ligands and are given in Table 2. The ligands are relatively average in size with molecular weight between 284.3 and 375.2 g mol^−1^. **TD12** is in lead-like space since its MW is < 300 g mol^−1^ whilst **TD18** and **TD34** are in drug-like space (for the definition of *lead-like*, *drug-like* and *Known Drug Space (KDS)* regions, readers are referred to ref. [48] and Table S1 in the Supplementary Material). A similar situation is seen for Log P, one ligand, **TD18**, is in the drug-like space with the other two in lead-like space. In general, the three hits are on the border between lead- and drug-like spaces with no value reaching the larger KDS. This is most likely due to the pre-filtering based on the molecular descriptors of the library prior to screening.

For further analysis, the Know Drug Indexes 2a and 2b (KDI_2a/2b_) [49] were also derived. The KDIs reflect the overall balance of the six molecular properties calculated based on the statistical distribution of KDS and derivation of an index for each descriptor. KDI_2a_ is an additive value with a maximum of 6.0 and KDI_2b_, where the indexes are multiplied, gives 1.0 as its maximum. The average for KDI_2a_ for known drugs is 4.08 (±1.27) and the hit compounds have considerably higher values. The KDI_2b_ version has an average of 0.18 (±0.20) for known drugs and all the ligands have considerable better scores, indicating excellent bioavailability of the ligands.

### 2.9. Molecular Modelling for Mechanistic Insights

**Figure 8:**
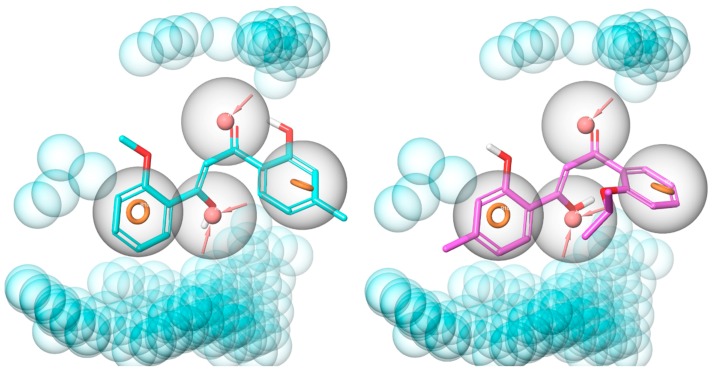
Alignment of **TD12** (cyan, left-hand side) and **TD18** (magenta, right-hand side) to pharmacophore model 2. The pharmacophore features are shown as orange rings (aromatic ring), pink spheres (H-bond acceptor), and blue spheres (excluded volumes); feature tolerances are showed as grey spheres around the pharmacophores.

The pharmacophore hypothesis used was made up of four features, two aromatic rings and two H-bond acceptors, as well as excluded volumes. **TD12** and **TD18** aligned well with these features avoiding the excluded volumes (Figure 8), demonstrating that the benzene rings, the hydroxyl and carbonyl oxygen atoms on the allyl linker are key pharmacophores. Interestingly, the enol tautomers of **TD12** and **TD18** were identified as active in the virtual screening cascade whereas their diketone forms scored poorly. The molecular modelling is therefore conducted on the enol tautomers. Furthermore, according to DFT calculations the enol form, of both **TD12** and **TD18**, is only 1.7 kcal mol^−1^ less stable than the diketone resulting in a considerable population of the enol.

In order to test this hypothesis and further understand the compounds’ interactions with the target enzymes, we performed QPLD (QM-Polarized Docking), which provides improved accuracy but at the expense of long calculation times when compared to the classical molecular mechanics. This is especially valid in the presence of transition metals by treating partial charges of ligand-receptor complex with quantum mechanics. This was evident with the RMSD values between the original conformation of the co-crystallized inhibitors and their docked conformation by extra precision Glide versus the QPLD approach. These values were 0.59 Å vs. 0.19 Å for 6A4I (TDO2), 0.68 Å vs. 0.42 Å for 5EK3 (IDO1), and vs. 0.53 Å vs. 0.37 Å for 6E43 (IDO1).

TDO2 is composed of four identical subunits each containing four α-helices and a heme cofactor catalysing oxidation of tryptophan to *N*-formylkynurenine. The iron at the centre of the heme is coordinated with six ligands: four with the pyrrole nitrogen atoms from the protoporphyrin; the fifth with the nitrogen atom of histidine (His328) side chain; and the sixth with an O_2_ molecule, substrate tryptophan or an inhibitor [50]. Dissociation of heme groups in heme-containing proteins is considered as the initial step of inhibition when ligands bind to apo forms of these proteins [51]. So far the 3D structure of only the holo human TDO2 has been identified. On the other hand, available crystal structures of apo IDO1 let us evaluate the affinity of the compounds to heme-free, as well as heme-containing IDO1.

**Figure 9:**
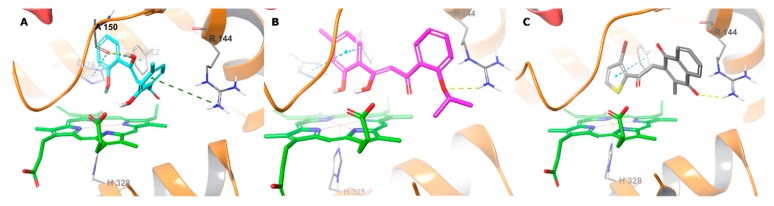
Binding interactions of **TD12** (**A**), **TD18** (**B**) and **TD34** (**C**) with holo human TDO2 active site residues according to molecular docking. Ligands and heme are shown as colour sticks, protein as ribbons, and binding interactions as coloured dashed lines, yellow for H-bond, blue for π–π, and green for π–cation.

**TD12** and **TD18** fit to the substrate-specific catalytic site of human TDO2 with high affinity (Table 3). We observed that the oxygen of 2-hydroxyphenyl of **TD12** and the oxygens of 2-isopropyloxyphenyl and hydroxyallyl of **TD18** were within 4 Å of the heme iron (Figure 9). The OH on the allyl linker of **TD12** also donated an H-bond to Ala150 side chain oxygen, which proves the key role of the tautomeric forms of these compounds in TDO2 inhibition. The compounds were stabilised in the catalytic site by key interactions with Arg144, Phe72 and His76 via their phenyl rings. The importance of these residues in the catalytic activity was proved in a mutagenesis study [52]. Some of these interactions were also detected for the co-crystallized inhibitor. These results support the pharmacophore hypothesis except the carbonyl oxygen as H-bond acceptor. In this QM-optimized docking the HOMO-LUMO gaps of the receptor complexes with **TD12** and **TD18** were found to be −0.098 and −0.085 hartree, respectively, showing that the inhibitors were stable in the catalytic site.

In the case of **TD34**, the 3-bromothiophene ring positioned perpendicular to the heme with the sulphur atom close to the iron. The ring is predicted to form π–π stacking with Phe72 and one of the oxygen atoms of the 2-methylnaphthalene-1,4-dione ring accepted H-bond from the Arg144 side chain (Figure 9C). However, the affinity of the **TD34**-6A41 complex was low. This could be due to weak interaction with the heme iron, as the low gap of HOMO-LUMO energies (−0.026 hartree) indicated lack of stability for this complex. Thus, **TD34** is expected to preferentially bind to the apo enzyme.

**Figure 10:**
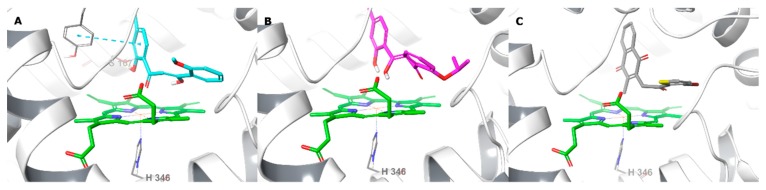
Binding interactions of **TD12** (**A**), **TD18** (**B**) and **TD34** (**C**) with holo human IDO1 active site residues according to molecular docking. Ligands and heme are shown as colour sticks, protein as ribbons, and π–π interactions as blue dashed lines.

Unlike TDO2, the catalytic site of IDO1 has an additional hydrophobic pocket thus can accommodate larger ligands [30]. The compounds, being relatively small, fit well in the catalytic site, without occupying the additional hydrophobic cavity (HOMO-LUMO gap: −0.103 and −0.099 hartree for **TD12** and **TD18**, respectively) and showing similar orientations as in the TDO2 active site. The oxygen atoms of 2-hydroxy-4-methylphenyl of **TD12** and **TD18** were close to the heme iron, as well as the oxygen of carbonyl of **TD12** and hydroxyl of **TD18** at the allyl linker, all together forming a triangle with the iron (Figure 10). In addition, **TD12** formed an H-bond via its aromatic hydroxyl with Ser167 and π–π staking with Tyr126, which were reported to take part in the electrostatic interactions network of co-crystallized inhibitors [51]. Although **TD34** showed affinity to the heme-containing IDO1, an effective interaction with the heme iron or nearby key residues was absent (Figure 10) and the HOMO-LUMO gap (-0.066 hartree) was quite small.

**Figure 11:**
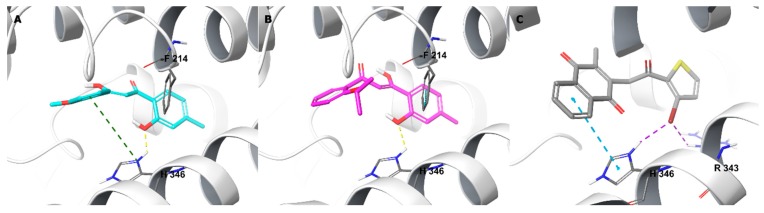
Binding interactions of **TD12** (**A**), **TD18** (**B**) and **TD34** (**C**) with apo human TDO2 active site residues according to molecular docking. Ligands and heme are showed as colour sticks, protein as ribbons, and binding interactions as colour dashed lines, yellow for H-bond, violet for halogen bond, blue for π–π, and green for π–cation.

Furthermore, **TD12** and **TD18** bound to the heme-free IDO1 active site with high affinity. Through the phenolic oxygen, they formed an H-bond with the His346 sidechain (Figure 11), which normally coordinates with the heme iron in the holo enzyme, indicating that both compounds may also have affinity to the apo IDO1. **TD34** bound strongly to the apo IDO1 by interacting with His346 side chain via the 2-methylnaphthalene-2,4-dion ring and the bromine moiety from both sides. The bromine also made electrostatic contacts with the Arg343 side chain, forming an array of interactions among **TD34** and the two residues, which was also the case with the co-crystallized inhibitors of heme-free IDO1 [32,51]. **TD34**, as a dual TDO2-IDO1 inhibitor, appears to have stronger affinity to the apo forms of these enzymes.

## 3. Materials and Methods

### 3.1. Virtual Screening

Compounds from the test set [32,33] and InterBioScreen Ltd. (IBS, Moscow, Russian Federation) [37] data bases were structurally optimised using MacroModel (2018-4: Schrödinger, LLC, New York City, NY, USA, 2018) with the OPLS_2005 force field [53]. The ligands were prepared using LigPrep module of Maestro (2018-4: Schrödinger, LLC, New York City, NY, 2018) by desalting, creating possible enantiomers, tautomeric and ionization states at pH 7±2. Their physicochemical descriptors were calculated using QikProp (version 3.2, New York City, NY, USA, 2018) [54]. The reliability of it is established for the calculated descriptors [55]. The crystal structures for human TDO2 (PDB ID: 6A4I, resolution 2.65 Å) and IDO1 (5EK3, resolution 2.21 Å [30] and 6E43, resolution 1.71 Å [31]) were downloaded from the Protein Data Bank [56,57] and prepared using Protein Preparation Wizard (2018-4: Schrödinger, LLC, New York City, NY, USA, 2018) [58]. Solvent and non-protein residues were removed, hydrogens were added, missing sidechains were filled with Prime (2018-4: Schrödinger, LLC, New York City, NY, USA, 2018), ionization and tautomeric states were generated by Epik (2018-4: Schrödinger, LLC, New York City, NY, USA, 2018), hydrogen orientations were set by Propka. Receptor grid maps were prepared using Receptor Grid Generation tool of Glide (2018-4: Schrödinger, LLC, New York City, NY, USA, 2018) by setting the central coordinates of the inhibitor for each pdb structure as the centre of search space. Each ligand of the test set was docked, keeping the ligands flexible to the grid, using Glide at extra precision 50 times [59,60,61]. Pharmacophore modelling was performed with Phase (2018-4: Schrödinger, LLC, New York City, NY, USA, 2018) using default settings [62,63]. For 3D shape similarity screening, Maestro’s Shape Screening panel was used. The active compounds from IBS library (**TD12**, **TD18** and **TD34**) were docked according to the QM-Polarized Docking (QPLD) protocol [64] of Maestro (2018-4: Schrödinger, LLC, New York City, NY, USA, 2018). The ligands were initially docked to the active site using extra precision Glide (five runs), QSite and Jaguar [65] (2018-4: Schrödinger, LLC, New York City, NY, 2018) were used to calculate single-point energies of each docking complex using the Density Functional Theory [66]. Partial atomic charges were derived using electrostatic potential fitting; the ligands were re-docked using Glide again at extra precision (ten runs) and the best poses were predicted according to Coulomb-van der Waals energies.

For the e-pharmacophore method, the crystal structure of the TDO2-inhibitor 1-(6-chloro-1*H*-indazol-4-yl)cyclohexan-1-ol (ligand ID: **9R9**) molecular scaffold was used for the pharmacophore query. 3D shape similarity screening was performed using Maestro’s Shape Screening panel with default settings.

In order to prepare the accumulation curves and determine the R_AUC_ values, the test set compounds were ranked according to their respective score from each method (shape similarity score for 3D shape similarity screen, Phase screen score for pharmacophore screen, and docking score for molecular docking). Then, the number of active compounds (as defined according to their biological activity data) among the top 34 scoring compounds was determined. Phase screen score is a scoring function that evaluates the quantity and quality of ligand feature matching, where quality is defined by site, vector, and volume scoring components.

### 3.2. Cell Based Kynurenine Assay and Measurement of Cellular Metabolic Activity

Breast cancer cell lines MCF7, MDA-MB-231, BT549, Hs578T; ovarian cell line SKOV3; glioblastoma cell line A172 and lung cancer cell line H460 were grown in α-MEMGibco, Grand Island, NY, USA; ThermoFisher catalogue number 12000022) containing 5% foetal calf serum (Moregate Biotech, Hamilton, New Zealand), insulin/transferrin/selenium supplement (Roche, Ref-11074547001), added according to the manufacturer’s instructions, as well as penicillin/streptomycin (100 U/mL and 100 μg/mL, respectively). The amount of kynurenine produced by the cells was measured as described previously [67]. Briefly, a well of a 96-well plate received 3.7 × 10^4^ cells which were subsequently cultured with the addition of 50 μM (BT474 and SKOV3 cells) or 100 μM (A172 cells) of L-tryptophan (Sigma-Aldrich, St Louis, MO, USA) with or without test compounds at 37 °C, 5% CO_2_ for 18 h. The cell number seeded was optimised to produce levels of kynurenine <40% of initial tryptophan to avoid tryptophan starvation and to ensure steady-state IDO1/TDO2 kinetics. Next, the culture supernatants were transferred into a fresh 96-well plate, incubated with trichloroacetic acid (10% final concentration) for 20 min at 60 °C to release kynurenine from the cells and precipitate proteins. After spinning the plates for 20 min at 2500 *g*, the supernatants were mixed at a 1:1 ratio with 4-(dimethylamino)benzaldehyde (20 mg/mL in acetic acid; Sigma) and the absorbance was read at 480 nm on an EnSpire 2300 Multimode Plate Reader (Perkin-Elmer, Singapore, Singapore). The remaining cells in the 96-well plate received a well-established indicator of cellular metabolic activity WST-1 (Roche Diagnostics Cat No 05015944, Basel, Switzerland; 10% diluted in culture media). After 1 h of incubation at 37 °C, the absorbance was measured at 440 nm [68]. WST-1 is reduced on the outer membrane of viable cells to a soluble formazan by reducing equivalents such as NAD(P)H. The absorbance measured at 440 nm indicates the number of metabolically active cells in the culture and represents a surrogate marker of cell viability.

As described previously [69], the sulforhodamine B colorimetric assay (SRB), which is based on the measurement of cellular protein content, was used to measure cell density [70]. After drug treatment for 3 d, cells were fixed with 10% (*w*/*v*) trichloroacetic acid and stained for 30 min, and the excess dye was removed by washing repeatedly with 1% (*v*/*v*) acetic acid. The protein-bound dye was dissolved in Tris base solution (10 mM) for optical density determination at 510 nm using a microplate reader. Optimal cell densities were previously determined to select initial cell densities that ensured that cells were in logarithmic phase for the experiments.

All experiments were repeated independently at least twice in technical quadruplicates. Calculations and graph plotting were performed in Prism (v8, 2018; GraphPad software, San Diego, CA, USA) and R computing environment (v3.5.3, 2019, 64-bit, Vienna, Austria). IC_50_ of the compounds in cellular enzymatic activity compared to untreated control cells was determined by fitting data to an equation *f(x)* = A + B/(1 + (IC_50_/X)^HILL) in Prism; where A is the minimum and B is the maximum of the curve, HILL is the slope of the curve and X is the compound concentration. 

### 3.3. Western Blotting

This procedure was carried out as described previously [69,71,72]. Breast cancer cell lines were grown to log-phase, washed twice with ice-cold PBS, and lysed in a SDS lysis buffer (60 mM Tris-HCl (pH 6.8 at 25 °C), 2% (*w*/*v*) SDS, 10% glycerol). Protein concentration was quantified using the bicinchoninic acid reagent (Sigma). Proteins (25 μg) were separated by SDS-polyacrylamide gel electrophoresis (PAGE), and transferred to PVDF membranes (Millipore Merck, Auckland, New Zealand). Membranes were immunoblotted with antibodies against human IDO1 and tubulin (Sigma). Bound antibody was visualized using Pierce™ ECL Western Blotting Substrate (ThermoFisher Scientific) and the chemiluminescence measured using image analyser LAS-3000 (Fujifilm, Tokyo, Japan). 

### 3.4. Density Functional Theory

The geometry optimisation and energy calculations were performed with Gaussian 16 [73] software using restricted DFT. The non-local B3LYP [74,75] functional hybrid method was employed and the standard 6-31+G(d, p) [76,77] diffused basis set was used for the geometry optimisation and frequency analysis vacuum. The zero-point vibrational energies (ZPE) were scaled according to Wong (0.9804) [78]. In all cases, the normal modes revealed no imaginary frequencies indicating that they represent minima on the potential energy surface. The subsequent energy calculations were performed with the larger 6-311+G(2df, p) basis set in vacuum. The ionisation potentials and electron affinities were calculated as described by Forseman and Frisch [79].

## 4. Conclusions

Ligand- and pharmacophore-based virtual screening was conducted against the heme containing enzymes IDO1 and TDO2. They catalyse the same rate-limiting step in the kynurenine pathway, an important biochemical mechanism for immunological response. ~6.7×10^4^ natural products were screened resulting in thirty virtual hits, which were tested in three cancer cell lines. These were glioma A172 only with TDO2 expression, ovarian SKOV3, which only expresses IDO1 and finally the breast cancer cell line BT549, which expresses both enzyme. Three active dual inhibitors were identified: **TD12**, **TD18** and **TD34**, with good kynurenine suppression with an IC_50_ of 3.42 µM for BT549 (**TD34**). Given the expression of both IDO1 and TDO2 in tumours, inhibiting only IDO1 is unlikely to generate desirable clinical outcomes. Hence, it can be argued that TDO2 needs to be inhibited as well as making dual inhibitors desirable for further development of drug candidates for inhibition of the immunosuppressive tryptophan catabolism mediated by IDO1/TDO2. **TD12** and **TD18** are close structural derivatives whereas **TD34** is a 1,4-quinone derivative. All have favourable physicochemical properties and are chemically tractable for further development.

## Figures and Tables

**Table 1:** R_AUC_ values of the accumulation curves for the query compounds obtained using the three modes of shape similarity screening.

Query	Typed Pharmacophore	Typed Atoms	Untyped Atoms
**1a**	0.76	0.95	0.95
**1b**	0.65	0.90	0.87
**1c**	0.78	0.96	0.97

**Table 2:** The molecular descriptors calculated for **TD012**, **TD018** and **TD34** as well as their Known Drug Indexes (KDIs).

	MW	PSA	#Rotor	DonorHB	AccptHB	Log P	KDI_2a/b_
**TD12**	284.3	73.3	6	1	4.5	2.7	5.70/0.75
**TD18**	312.4	72.2	7	1	4.5	3.5	5.67/0.71
**TD34**	375.2	76.0	3	0	6.0	2.6	5.69/0.70

MW: Molecular weight. PSA: Van der Waals surface area of polar nitrogen and oxygen atoms and carbonyl carbon atoms. #rotor: number of rotatable bonds. donorHB: H bond donor count. accptHB: H bond acceptor count. Log P: Predicted octanol/water partition coefficient. KDI: Known Drug Index.

**Table 3:** Docking scores (kcal/mol) * for the hit matter.

Ligand	6A4I	5EK3	6E43
**TD12**	−7.9	−6.4	−7.2
**TD18**	−7.4	−6.8	−7.4
**TD34**	−3.9	−6.3	−8.2

* XPGscore from the QM-optimized re-docking.

## Supplementary Materials

The following are available online, Figure S1. Structure of the active and inactive compounds of the test set, Figure S2. The thirty structures of the virtual hits (**TD01** to **TD30**), which were tested for activity, Table S1. Definition of lead-like, drug-like and Known drug space (KDS) in terms of molecular descriptors, Table S2. The catalogue numbers of the commercial compounds tested and their source, Table S3. The single point and corrected zero point vibrational energies (ZPE) of the optimized ligands in hartrees (a.u.).
