# Supplementary material for: Discovery and Characterisation of Dual Inhibitors of Tryptophan 2,3-Dioxygenase (TDO2) and Indoleamine 2,3-Dioxygenase 1 (IDO1) Using Virtual Screening
Source: Molecules. 2019 Nov 28;24(23):4346. doi: 10.3390/molecules24234346 (PMC6930675; doi:10.3390/molecules24234346)
Supplement: Supplementary file 1 [file molecules-24-04346-s001.pdf]

Supplementary Material

Discovery and characterisation of dual inhibitors of tryptophan 2,3-dioxygenase (TDO2) and indoleamine 2,3-dioxygenase 1 (IDO1) using virtual screening

Suat Sari [1], Petr Tomek [2], Euphemia Leung [2,*] and Jóhannes Reynisson [3,*]

[1] Hacettepe University, Faculty of Pharmacy, Department of Pharmaceutical Chemistry, Ankara 06100, Turkey; suat1039@gmail.com

[2] Auckland Cancer Society Research Centre, University of Auckland, Grafton, 1023 Auckland, New Zealand; p.tomek@auckland.ac.nz

[3] School of Pharmacy and Bioengineering, Keele University, Hornbeam building, ST5 5BG Staffordshire, United Kingdom

* Correspondence: e.leung@auckland.ac.nz (E.L.); j.reynisson@keele.ac.uk (J.R.); Tel.: +44(0)1782733985 (J.R.)

Content

1. **Figure S1.** Structure of the active and inactive compounds of the test set…………………………….…..2
2. **Figure S2.** The thirty structures of the virtual hits (**TD01** to **TD30**), which were tested for activity………3
3. **Figure S3.** The five derivatives of **TD05** (**TD31** to **TD35**), which were tested for activity……………...……4
4. **Table S1**. Definition of lead-like, drug-like and Known drug space (KDS) in terms of molecular descriptors. ………4
5. **Table S2**. The catalogue numbers of the commercial compounds tested and their source…………………..5
6. **Table S3.** The single point and corrected zero point vibrational energies (ZPE) of the optimized ligands in hartrees (a.u.)……………………………………………………………………………………………………6

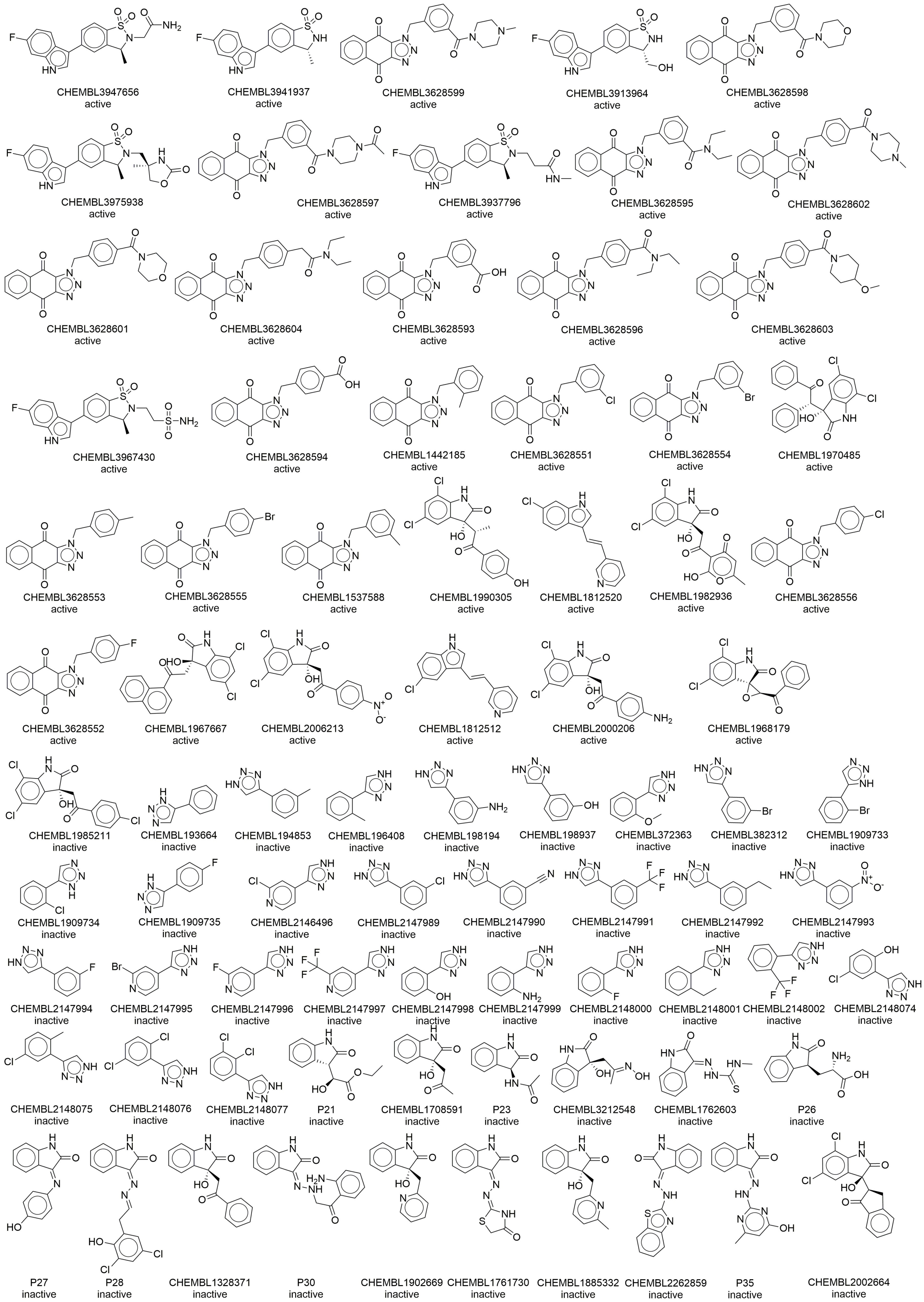

Figure S1. Structure of the active and inactive compounds of the test set (P21, P23, P26 P27, P28, P30, and P35 were obtained from the study of Pantouris et al. 2016).

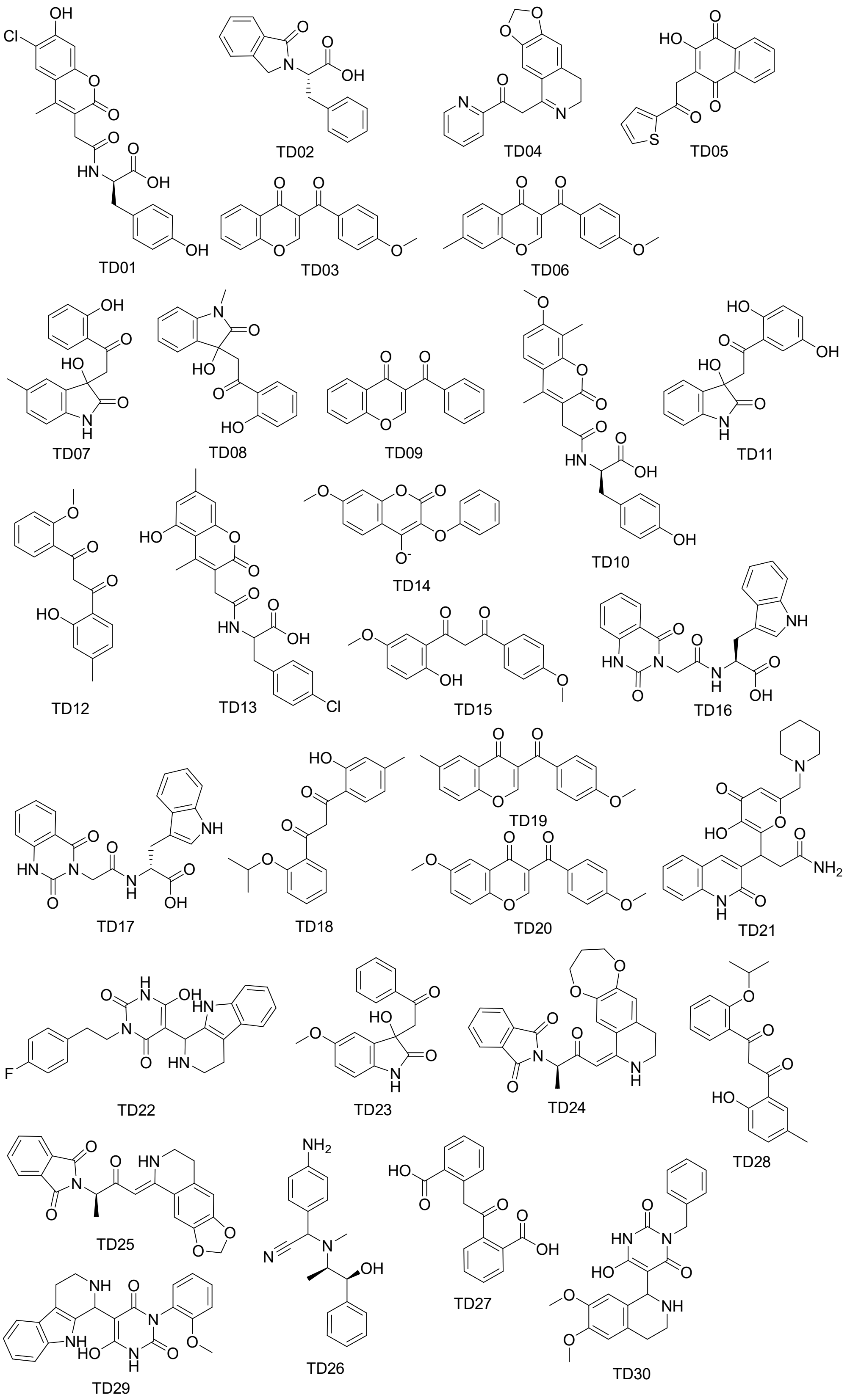

Figure S2. The thirty structures of the virtual hits (**TD01** to **TD30**), which were tested for activity.

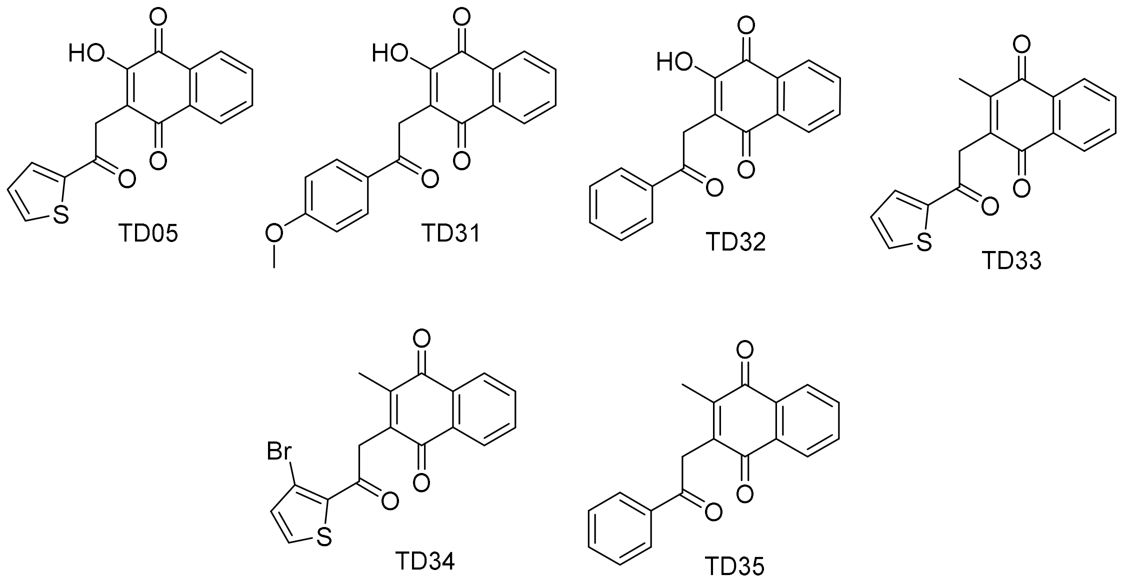

Figure S3. The five derivatives of **TD05** (**TD31** to **TD35**), which were tested for activity.

Table S1. Definition of lead-like, drug-like and Known drug space (KDS) in terms of molecular descriptors. The values given are the maxima for each descriptor for the volumes of chemical space used.

	**Lead-like Space**	**Drug-like Space**	**Known Drug Space**
Molecular weight (g mol^{-1})	300	500	800
Lipophilicity (Log P)	3	5	6.5
Hydrogen bond donors (HD)	3	5	7
Hydrogen bond acceptors (HA)	3	10	15
Polar surface area ($Å^2$) (PSA)	60	140	180
Rotatable bonds (RB)	3	10	17

Table S2. The catalogue numbers of the commercial compounds tested and their source.

TDO code	compound name	Supplier
TD_001	STOCK1N-52742	InterBioScreen
TD_002	STOCK1N-74210	InterBioScreen
TD_003	STOCK1N-19585	InterBioScreen
TD_004	STOCK1N-65400	InterBioScreen
TD_005	STOCK1N-45023	InterBioScreen
TD_006	STOCK1N-88445	InterBioScreen
TD_007	STOCK1N-29263	InterBioScreen
TD_008	STOCK1N-28512	InterBioScreen
TD_009	STOCK1N-20765	InterBioScreen
TD_010	STOCK1N-41695	InterBioScreen
TD_011	STOCK1N-14647	InterBioScreen
**TD_012**	STOCK1N-83399	InterBioScreen
TD_013	STOCK1N-51797	InterBioScreen
TD_014	STOCK1N-09393	InterBioScreen
TD_015	STOCK1N-21184	InterBioScreen
TD_016	STOCK1N-57094	InterBioScreen
TD_017	STOCK1N-66050	InterBioScreen
**TD_018**	STOCK1N-82503	InterBioScreen
TD_019	STOCK1N-20871	InterBioScreen
TD_020	STOCK1N-89459	InterBioScreen
TD_021	STOCK1N-90415	InterBioScreen
TD_022	STOCK1N-55924	InterBioScreen
TD_023	STOCK1N-89920	InterBioScreen
TD_024	STOCK1N-88688	InterBioScreen
TD_025	STOCK1N-89918	InterBioScreen
TD_026	STOCK1N-27918	InterBioScreen
TD_027	STOCK1N-09308	InterBioScreen
TD_028	STOCK1N-77813	InterBioScreen
TD_029	STOCK1N-57965	InterBioScreen
TD_030	STOCK1N-31554	InterBioScreen
TD_031	5169036	ChemBridge
TD_032	5477691	ChemBridge
TD_033	11K-929	Key Organics
**TD_034**	11K-930	Key Organics
TD_035	10E-980	Key Organics

Table S3. The single point and corrected zero point vibrational energies (ZPE) of the optimized ligands in hartrees (a.u.).

Systems	Energy/a.u.	ZPE/a.u. [a]
TD12	−958.6329072	0.286727784
Ox.	−958.3566669	0.286534645
Red.	−958.6515898	0.282028727
Enol taut.	−958.6305058	0.287089552
TD18	−1037.292005	0.34149783
Ox.	−1037.017137	0.341247828
Red.	−1037.30965	0.336714458
Enol taut.	−1037.289583	0.341853715
TD34	−3852.740195	0.231744011
Ox.	−3852.427131	0.228364572
Red.	−3852.810506	0.229085166

[a] Corrected values (Wong).
